# In-Door Amusement

**Published:** 1872-02

**Authors:** 


### IN-DOOR AMUSEMENT.

One of the most eminent of England's judges once said that a very large majority of criminals of all grades brought before him were found to have their characters fixed between the ages of eight and sixteen, in the street, after night. From eight to sixteen, from sundown to bed-time, keep your children out of the streets, keep them in the house, make home pleasant, amusing, entertaining, delightful. Did ever such a home turn out a worthless son, a ruined daughter? Who can answer in the affirmative? Then a weighty responsibility rests on parents to provide such a home, to make it more inviting than the street, more inviting than the homes of neighbors and friends, more inviting than the ball-room, the theatre, and the dance. A step has been taken in the right direction by one of our most enterprising publishing houses, Lee and Shepard, in printing a book of 380 pages, 12mo, in most attractive binding, called "The American Home Book of In-door Games, Amusements and Occupations," by Mrs. Caroline L. Smith. Illustrated. Sent post paid for about a dollar and a half. It contains amusements for little girls; another set of amusements for little boys; then games for little children; also musical games for little children; games for children from five to twelve; also forfeits and games of memory; games for old and young; new games; natural magic, for Christmas, for New Year's; ventriloquism; gardening; flowers; house and home arts; the toilet; home reading; excellent directions for the school-room; for making bread; with suggestions on politeness. A hundred thousand of these books ought to be sent to as many families, who are so blessed as to have children growing up; and many thanks to the thoughtful and kind hearted woman who has accomplished a work so timely, so useful, and so good.

